# Indents

**Published:** 1915-03

**Authors:** 


### INDENTS.

☞ **Brief Articles of Technical or Scientific Value will receive notice and comment. Contributions invited.**

*Errors in Diagnosis.* Since the discovery of a positive relationship between tonsil infections and secondary manifestations in other organs and parts, tonsils have been removed wholesale, in some instances on slight provocation. Their removal has, we fear, become with some almost a fad. A similar fad is growing up relative to the removal of teeth, since the discovery that jaw abscesses and pyorrhea alveolaris are equally as potent foci for secondary infections as are the tonsils. Some physicians are rather indiscriminately sending their patients to the extraction specialist, requiring removal of several or all of the teeth, supposing them to be a factor in some lesion, when their removal is not always justified. A Roentgenogram, if properly made, is infallible, and can be absolutely relied on providing its interpretation is correct, but an improper exposure may lead one, if inexperienced, to mistake a part of the maxillary sinus for an abscess at the end of the roots of the second bicuspid or first molar. Other errors from incorrect readings may lead to similar wrong conclusions.—*T. L. Gilmer in Summary*, p. 20, 1915.

*Autogenous Vaccines.* Regarding autogenous vaccines, our ideas need to be revised somewhat in view of the fact that the vaccines administered are usually prepared from streptococci in the focus which may or may not be the causative organism. The instances in which good results are obtained are probably those in which the focus harbored the causative organism of the vaccine prepared from it. The instances in which poor results are obtained may be due to the fact that the vaccine does not contain the proper antigen.

With regard to the foci: What are they, where are they, and what organisms are usually present? Probably the most common location of the focus or source of infection is the mouth. The tooth socket has a structure similar to that of the joints, and a blind abscess at the apex of a tooth may have no more significance than the lesion in the joint. While it is proper to remove the teeth that are abscessed in a case of that kind, we should not expect that that is necessarily the source of the trouble, but must look still further.—*Dr. E. C. Rosenow in Journal A. M. A.*

*Amalgam as a Filling Material.* An ounce of some alloys for dental purposes isn't worth anything; for if your material is fundamentally wrong, the operation is of necessity a failure. An inferior alloy makes an inferior amalgam; an inferior amalgam makes an inferior restoration, no matter how you may wrestle with it; an inferior restoration commands an inferior fee and helps to establish an inferior reputation.

*Now over fifty per cent. of all the alloy used by American dentists isn't fit for dental purposes.*

That is a rather bold statement, but it can be established. There are two reasons for this demand for inferior alloys. The first is that so many dentists are accustomed to charge small fees for amalgam fillings, and therefore try to economize in their purchase of materials. The second is that the inferior alloys almost without exception are easier to work. Being composed largely of tin, they are very smooth and plastic when amalgamated; they are simply plastered into the cavity and the work is done.

Another reason that inferior alloys are used is that many of the dental colleges for reason of economy, use low-grade alloys. The result is that students grow familiar with these materials and afterwards retain them in their practice.—*Dental Quarterly.*

*Adhesion and Cohesion of Cements.* The molecular attraction exerted between the surfaces of bodies in contact is called adhesion. The force of adhesion may operate between solids, between liquids and solids, and between solids and gases.

Cohesion is the force which unites adjacent molecules of the same nature; for example, two molecules of water, or two molecules of iron. Cohesion is strongly exerted in solids, less strongly in liquids and scarcely at all in gases.

Adhesion and cohesion of dental cements can only be determined by physical methods. Adhesion between two surfaces is complete when the pores and crevices of the surface are filled with the cementing substance. In some cases the adhesion of cemented objects is so powerful that the mass breaks more readily at the uncemented parts. Contrary to prevalent opinion, the cement layer should be thin.

In most cases the advertiser of dental cements plays upon the appearance and consistency of the "cement mix on the slab" as an indication of its adhesive or cohesive qualities. The plastic mass of cement is continually undergoing chemical change so that the "set" mass is entirely different in character than the plastic mass. It naturally follows that the "smoothness of mix" has no relation to the ultimate physical properties of a dental cement. In many cases inferior dental cements are exceptionally smooth in the mix and as a matter of fact, all of the best cements are also smooth in the mix. This is merely a temporary physical property and is therefore only of corresponding importance when individually considered.—*Dental Quarterly.*

*Dental Foci of Infection.* Certainly enough is known concerning infections and their mode of entrance, that the infected and diseased mouth and respiratory tract must be looked on as most serious menaces. Much may be done by more general and effective school inspection. The present generation of children will understand and demand protection for their children in time. The first teeth should be watched, that the second be not permitted to erupt irregularly, causing deformities. Jaws should be spread that the teeth may meet and the high arched palate, diminishing nasal breathing, thereby reduced. Tonsils and adenoids should be looked after, thus preventing ear and mastoid diseases, rheumatism, endocarditis, etc. In chronic and recurring diseases, a search must be made to establish positively the non-participation of each of the several sources of infection.

The physicians engaged in this line of observation require fully as much training in the rudiments of dentistry as the dentist does in the signs of infectious diseases. While we have leaders in all professions, through the energy of their kinetic glands, the big stick which leads to our advancement is in the hands of the progressive and educated public who are constantly demanding more of their dentists, of the medical profession, and of the state in protecting them against preventable diseases.—*C. H. Mayo in Journal A. M. A.*

*Infective Foci in the Mouth.* We have in the mouth two groups of chronic foci; the chronic alveolar abscess and the so-called pyorrhea pocket, both of which involve a single tissue—the peridental membrane—which is the important factor in establishing the chronicity of both groups of cases. As a result of the involvment of the peridental membrane, either at the apex of the root or beginning at the gingival margin, there is a suppurative destruction of the attachment of the tissue to the cementum covering the root of the tooth. As a part of this process the cementoblasts, which overlie the surface of the cementum within the peridental membrane, are also destroyed. These are the only cells which could cause reattachment of that tissue. Soon after detachment, the fibers of the peridental membrane, which pass from the cementum of the detached area to the bone, disappear, and a little later the bone to which they were attached is absorbed. Thus all of the specialized elements necessary to the connection of root with bone are lost, and we can not have reattachment of this tissue to the cementum of the root. This is shown very clearly by the Roentgenogram in both groups of cases. Careful examination of these will show a line down the side of and close against the root, which is practically always in advance of destruction of the tissue elsewhere, showing clearly that this disease has begun at the junction of the peridental membrane and the cementum. There is a progressive involvement of peridental membrane and bone, making the whole area about a given tooth funnelshaped. Within the past few years I have cut away the tissue forming these pockets from the roots of teeth for microscopic examination. In one case I removed the tooth, peridental membrane and a section of the alveolar process and overlying gum tissue all in one piece, and had sections made for study. These sections show that the above mentioned changes take place and that detachments are permanent. Therefore pockets remain about such teeth and the overlying tissue is subject to frequent reinfection. We should not, therefore, expect vaccines to have any more than temporary effect on the local focus, because we have a condition here in which the physiologic possibility of repair is gone. The dental treatment indicated is the elimination of the focus in the case of the alveolar abscess by resecting the denuded root end, or the extraction of the tooth. If it is a pocket alongside the root, exposed to the fluids of the mouth, the tooth must be extracted, or palliative treatment employed which will be effective against reinfections.—*Journal A. M. A.*

*Arthritis Deformans.* Partial or complete ischemia of the tissues due to the embolism is an important factor in the production of the morbid anatomic changes. Oxhausen of Berlin has produced, in animals, aseptic osteochondritis, resembling arthritis deformans, by ligating the arteries supplying the joint tissues. These principles are, I think, susceptible of proof that a chronic alveolar infection, and chronic foci in other regions also, may cause systemic disease by hematogenous bacterial emboli, which infect and at the same time deprive the tissues of nourishment. Local infection of muscles, joint tissues, etc., and lessened blood-supply result in the peculiar morbid anatomy of the respective tissues.

To investigate and manage these patients requires team work of the clinical and laboratory workers. The clinician must carefully examine the patient, exhausting every detail in the personal history. The skill of the dentist, the nose and throat specialist, the gynecologist, the genitourinary expert and others may be necessary to locate the foci of infection. The focus must be destroyed. Tissues and exudates of foci should be carefully examined and bacterial cultures made. Vaccines of the dominant bacteria may be made for subsequent use.

With the source of the infection removed, the attempt should be made to increase the defenses of the body against the systemic infection. This involves a long and tedious period in the chronic joint infections and also in some other systemic diseases of focal origin. Rest, both mental and physical, is essential. Good, wholesome food, pure air, optimistic surroundings and, if necessary, restorative tonics of iron and other drugs. Pain must be palliated by simple drugs like the salicylates. After a time passive exercise and later active graduated exercise must be followed to aid in the restoration of local circulation and to hasten restoration of the morbid changes of the tissues. Autogenous vaccines may be used in the attempt to improve the defenses of the body. In chronic arthritis, with the circulation of the infected tissues obstructed embolically, antibodies in the blood stream, even if augmented by vaccines, would have but little effect locally. In the later stages of chronic arthritis when the local circulation has been so improved that the tissues are flooded with blood, vaccines will be of undoubted value. Patients may, however, be cured of chronic arthritis by the other measures named without the use of vaccines.—*Journal A. M. A.*

*Mixing Amalgam.* The directions presented are recommended as the best procedure for *all* high-grade alloys, with the exception that different alloys require different proportions of alloy and mercury. The proper proportions may be found in the printed directions supplied by the manufacturer.

If the operator will learn to use *only such excess mercury as is necessary to make a perfect mix,* such procedure will result in the ideal condition of plasticity of the amalgam essential to the most perfect and stable adaptation, avoiding the necessity of adding or removing mercury during the mixing, which is very inaccurate and uncertain, and will be found a common cause of those bulk changes which sometimes follow the use of our most reliable alloys.

Insufficient time or rapidity of movement of the pestle in the mixing will have the same result: namely, an imperfect mix, which will result in an unstable filling.

To insure accuracy: At convenient times the operator, or assistant, can weigh into capsules ten and fifteen grains of alloy, and into other capsules the necessary proportions of mercury. These capsules are ready for immediate use, without inconvenience or loss of time, and the use of alloy in definite quantities will result in a great saving of material, because the operator soon learns the amount required for any particular cavity.

All dental alloys require a slight excess of mercury to make a perfect mix, but such excess should not be so great as to make a mass that fails to retain its form when made into a roll after mixing for two and a half or three minutes; nor should the mass show any signs of crepitus or setting during the kneading or while packing the first half of the filling, as fillings made under such conditions will show faulty adaptation to cavity walls.—W. E. Harper, in *Review.*

*Teeth a Menace to Health.* It may seem incongruous or out of harmony with the present theories of dentistry to say that teeth as commonly found in the human mouth are a menace to both health and longevity, or to put the same truth in another way, to say that the loss of all teeth from the uncared for mouth in adult life would be a safeguard against many systemic diseases and markedly lengthen the average of human life. —D. D. Smith, in *Review.*

*Sanitary Bridge Work.* That the ordinary fixed bridge, carelessly planned and constructed, is open to criticism from the hygienic standpoint, is conceded. It is the writer's opinion, however, that selected cases, properly constructed, can be made to conform to hygienic demand, provided they are given the necessary attention by the patient. Every person wearing a fixed bridge is entitled to know the importance of maintaining its hygienic integrity, for the purpose of prolonging its length of service and, incidentally, their own good health. There are three other points in bridge construction which must be observed if we hope to attain anything approaching hygienic requirements, which are, the width of the cusps bucco-lingually, the width of embrasures, and the form of the solder connection between abutments and dummies. I am aware of the objection by many to a narrowing of dummy cusps bucco-lingually, but I maintain that they should never be made wide enough to form a "shelf" which can not be brushed clean with the tooth brush. The embrasures and interproximal spaces in fixed bridges should be sufficiently broad that they also may be cleaned in the act of brushing the teeth. The point of union between abutments and dummies should be but little larger than a normal contact point. Aside from the abutments, this junction point as it is commonly made is responsible for much of the criticism directed toward the fixed bridge by profession and laity. It is one of the most abused and neglected places in the entire bridge, and the most fruitful source of unsanitary and unhygienic manifestations. Careless construction at this point not only endangers the permanency of the abutment, but in some instances it threatens the health, happiness and frequently the very existence of the individual. Every means should be employed to make this part of the bridge meet the hygienic demands.

Wide embrasures and small contact points will eliminate these objections sufficiently to make the work practical. A small surface at this junction point can be obtained by the use of iridio-platinum wire for reinforcement, which gives ample strength without unnecessary bulk.—L. W. Strong, in *Dental Review.*

*Swaged Cast Plates.* The impression is made and the cast made in Spence's Plaster Compound. For a cast aluminum base the wax is carefully applied to the Spence Plaster Compound Cast, with rim, retainers, etc., and then half invested. This stiffens the wax in such a manner that it may be removed without distortion. The investment is then completed and the piece cast in aluminum. This casting being, from the inherent nature of the process, more or less inaccurate, the cast is placed on the original Spence Plaster Compound cast (in the meantime bedded in a ring supplied with the Ash press for the purpose) and the whole placed in the Ash swager, and the maximum pressure of the rubber blocks and finely divided rubber (next the model) applied producing a base, which, in the estimation of the speaker, is much superior to vulcanite and quite as accurate in fit and consequently adhesion, and little additional trouble. A soft metal ''lift'' may be placed between the casting and the Spence Plaster Compound cast before swaging for compensation of unusually hard areas in the median line. The technique for swaged metal base is very similar, the Spence Plaster Compound cast being used as a pattern from which the zinc or babbit cast is produced, and upon which the rough swaging is done, and the retaining wire, rim, etc., having been soldered, the whole is placed in the Ash press and reswaged similar to the cast aluminum process hereinbefore mentioned.—Dr. W. E. Cummer, in *Oral Health.*

*Local Treatment of Pyorrhea.* This is surgical and conducted according to surgical principles; with hands and instruments sterile. The actual work is to be preceded by a thorough spraying of the tissues of the mouth with physiological salt solution, followed by an equally thorough removal of the calcareous deposits, together with affected tissue and any alveolar tissue that is necrotic. Blind abscesses demand particular attention, and in certain cases destroying and removing the pulp of a tooth acts beneficially. Loose teeth should be stayed, cervical fillings made smooth and polished, other fillings properly contoured, in order that food might not pack between the teeth, irritating their roots by wedging and the gums by pressure, to say nothing of the elements formed from putrefaction of the impacted food mass, some of which, as ammonia, cause a direct precipitation of lime salts. The articulation of the teeth is most important in this connection, as force constantly exerted in an unnatural manner produces a condition of tissue particularly favorable to calcium deposition; and so, too, should all missing teeth be supplied, that the teeth may be properly supported and the articular surfaces restored as closely as possible. These are all familiar things and matters of everyday practice, but demand especial attention in an endeavor to rectify or to fend off this disturbance. Further, an examination should be made of the adjacent anatomy. Catarrh or sinus affections, by setting up an inflammatory condition of the uvula, or tonsilar affection, produce morbid substances that being reflected more or less into the mouth are capable of causing calcareous deposition. These facts should receive appropriate attention.

With respect to medication, following the surgical treatment or as an adjunct to it, the familiar astringent, counter-irritant, or caustic remedies are all of more or less value, and may be employed at the discretion of the operator. Dr. Head's ammonium bi-fluoride deserves a place in our list. As a rule, I believe that physiological salt solution most perfectly meets our present ideals of the physiological principles involved in healing tissue and I use it freely, and even inject it or Ringer's solution in certain cases, with a view to washing out the congested vessels. As a dentifrice, I prefer to have the patient use an alcoholic soap solution instead of a powder or paste, and instruct them to use a quantity of physiological salt solution for a rinse; filling the mouth and forcing the solution between and about the teeth and tissues with vigor. Massage of the gum conscientiously followed up, has a restorative effect upon the circulation of the soft tissues.—Percy R. Howe, in *Journal of Allied Societies.*

